# Skeletochronology, Body Growth and Effectiveness of Growth Marks to Estimate the Ages of Sumatran Water Monitor Lizards (*Varanus salvator macromaculatus*)

**DOI:** 10.21315/tlsr2025.36.3.4

**Published:** 2025-10-31

**Authors:** Hellen Kurniati, Ni Luh Putu Rischa Phadmacanty, Gono Semiadi, Wahyu Trilaksono, Fatahul Azwar

**Affiliations:** 1 Research Center for Biosystematics and Evolution, National Research and Innovation Agency (BRIN), Jalan Raya Cibinong (Jakarta-Bogor) km 46, Cibinong 16911, Indonesia; 2 Research Center for Applied Zoology, National Research and Innovation Agency (BRIN), Jalan Raya Cibinong (Jakarta-Bogor) km 46, Cibinong 16911, Indonesia; 3 Directorate of Scientific Collections Management, National Research and Innovation Agency (BRIN), Jalan Raya Cibinong (Jakarta-Bogor) km 46, Cibinong 16911, Indonesia; 4 Research Center for Ecology and Ethnobiology, National Research and Innovation Agency (BRIN), Jalan Raya Cibinong (Jakarta-Bogor) km 46, Cibinong 16911, Indonesia

**Keywords:** Sumatra, Common Water Monitor Lizards, *Varanus salvator macromaculatus*, Age, Skeletochronology

## Abstract

Demand for water monitor lizards (*Varanus salvator macromaculatus*) from Sumatra and Kalimantan for the leather industry is the highest compared to varanid species from Java (*V. s. bivittatus*) or Sulawesi (*V. s. ziegleri*). No conclusive evidence on the age estimate of the individuals being harvested. The skeletochronology method was used to estimate harvested Sumatran water monitors’ age and body growth using fibular bones. The mid-diaphysis fibula bone cross-sections of 81 individuals (39 females and 42 males) showed that the line of arrested growth (LAG) was not always clearly visible. Periosteal bones in individuals with SVLs of 39.0 cm–70.0 cm showed double and multiple LAGs and only a few individuals had apparent LAG. As the SVL increases, the double and multiple LAGs are not seen in individuals with more than 70 cm SVL. The estimated ages of harvested individuals of female *V. s. macromaculatus* with SVLs ranging from 40.1 cm–71.0 cm were 2–3 years old, whilst for males with SVLs ranging between 39.0 cm–96.0 cm, were 2–5 years old. A strong correlation between the SVL and fibular mid-diaphysis diameter was observed in both sexes. This indicates that the formation of LAGs occurs steadily every year, although LAGs are not always clearly visible in every individual, especially in young ones.

**HIGHLIGHTS**

- The mid-diaphysis fibula bone cross-sections of Water Monitor Lizards (*Varanus salvator macromaculatus*) individuals showed that the line of arrested growth (LAG) was not always clearly visible.
- The estimated ages of harvested individuals of female *V. s. macromaculatus* with SVLs ranging from 40.1 cm–71.0 cm were 2–3 years old, whilst for males with SVLs ranging between 39.0 cm–96.0 cm were 2–5 years old.
- A strong correlation between the SVL and fibular mid-diaphysis diameter was observed in both sexes.

## INTRODUCTION

The common Asian water monitor lizard, *Varanus salvator* is widely distributed in Indonesia, stretching from Sumatra, Java, Kalimantan and Sulawesi to their satellite islands, the Lesser Sunda Islands, and several islands in the Moluccas (Quah *et al*. 2021). It is a large varanid lizard with a snout-to-vent length (SVL) of more than 2 m (Bennet 1998). According to Auliya and Koch (2020), there are four subspecies of *V. salvator* in Indonesia: *V. s. macromaculatus* in Sumatra and Kalimantan; *V. s. bivittatus* in Java and adjacent islands; *V. s. ziegleri* in Obi Island; and V. s. celebensis in North Sulawesi.

The demand for common water monitor lizards from Sumatra and Kalimantan for the leather industry is higher than that for varanid species from Java or Sulawesi for their skin thickness and quality. Most of the leather is exported to Europe and there are no size limitations for individuals harvested for their skin; therefore, juvenile and fully mature individuals are used (Shine *et al*. 1996; 1998). Approximately 95% of common water monitor lizard pets are juvenile (Kurniati *et al*. 2023). In Indonesia, harvesting from the wild for commercial purposes is controlled through a quota mechanism set by CITES, as the species is listed in Appendix II (CITES 2023).

The habitats of common water monitor lizards are diverse, including primary forests, secondary forests, palm oil plantations, mangrove forests, brackish and freshwater swamps, and human settlements in rural and urban areas (Bennett 1998; Das 2004; Khadiejah *et al*. 2020). Common water monitor lizards were found to be more abundant in modified habitats in rural and urban areas than in natural forests (Khadiejah *et al*. 2020; Shine *et al*. 1996; 1998; Uyeda 2009). Most hunters in Sumatra captured the lizards along large rivers and freshwater swamps close to a large river, rainforest area or inside palm oil plantations (Kurniati & Riyanto 2008; Shine *et al*. 1996).

Information regarding age correlation with body size in common water monitor lizards is limited. The body growth measurements intended to determine the correlation with the age of *V. salvator* were carried out by Andrews (1995) using captive individuals and growth measurements in the wild were done by Bin Abdul and Bin Abdullah (1988). However, the application of the age estimator is still limited.

Age-estimation studies of varanids using the skeletochronology method have been conducted on *V. griseus* (Smirina & Tsellarius 1996) and *V. niloticus* (De Buffrenil & Castanet 2000). A study on the skeletochronology of long tubular bones in juvenile *V. salvator* was conducted by Kurniati *et al*. (2023). It shows that the fibula was the best predictor for age estimation through the Line of Arrested Growth (LAG), as demonstrated also in *V. niloticus* (De Buffrenil & Castanet 2000). However, no conclusive evidence was found with *V. s. macromaculatus*. This study aimed to determine the age estimation of *V. s. macromaculatus* and test whether the skeletochronology method is applicable to this species.

## MATERIALS AND METHODS

### Ethics Statement

The study was conducted in a large collector premises that acted as the slaughterhouse in Palembang, South Sumatra, in June 2022. No individuals of Sumatran common water monitor lizards (*Varanus salvator macromaculatus*) were intentionally killed for this study. Ethical approval was obtained from the National Research and Innovation Agency, Republic of Indonesia (No. 138/KE.02/SK/07/2022). All limb bone examinations of the fibula were performed after the animals were killed, skinned and discharged as waste products by the slaughterhouse.

### Specimens

Eighty-one individuals (39 males and 42 females) were used in this study. Each dead individual was randomly selected, and sex was identified according to secondary sexual characteristics. The characteristics of the reproductive stage, as immature or mature, were determined according to Shine *et al*. (1996). The snout-to-vent length (SVL) was measured using polypropylene tape and followed by dissection of the belly to examine the reproductive status.

### Skeletochronology

Skeletochronology was performed following the method used by De Buffrenil and Castanet (2000), with the steps of fibula bone processing following Sinsch (2015). The fibula bones were decalcified using 10% formic acid (Merck, USA) for 72 h–120 h, according to their size. Once the bone had softened, it was cut at the diaphysis approximately 1 cm in length using a scalpel and stained with Erlich’s hematoxylin for 8 h–24 h. The specimens were then cut transversally in 20 μm thickness using a freezing microtome (Yamato-RV 240, Japan; Comas *et al*. 2016). At least three good cross-section specimens were selected at the mid-diaphyseal part with the smallest marrow cavity for each fibula bone. All cross-sectioned fibular bones were mounted in entellan mounting media (Merck, USA) and analysed using a compound microscope (Olympus, CX43, Japan) at 20× magnification which attached to a computer photograph. The visible Line of Arrested Growth (LAG) was counted and the longest diameter (D) was measured using ImageJ version 1.53 (Schneider *et al*. 2012).

### Data Analysis

The PAST software (Hammer *et al*. 2001) was used to analyse the data for regression between SVL and D. A one-way ANOVA was performed to test sexual dimorphism in bone size.

## RESULTS

**FIGURE 1:**
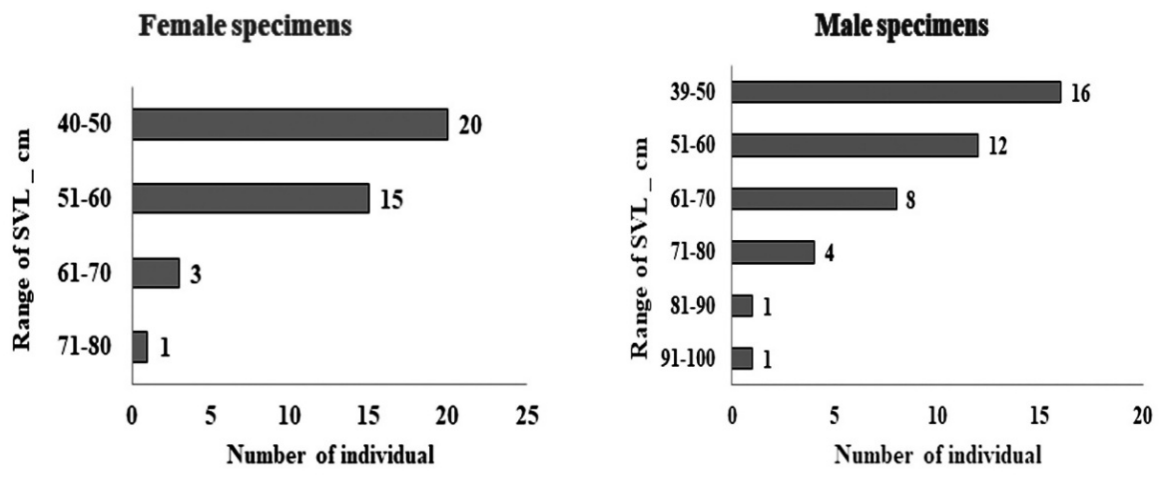
Number of individuals which limb bones were collected and their SVL range.

The range of SVL for females was 40.1 cm to 71.0 cm, and males were 39.0 cm to 96.0 cm (Fig. 1). The most frequently hunted individuals across the sexes had an SVL of 51 cm to 60 cm.

**FIGURE 2:**
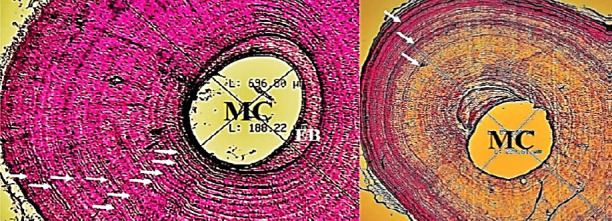
Double or multiple LAG in a female bone cross-section with an SVL of 45 cm (left, white arrow: LAG). Visible LAG formation formed from two LAGs in a female with an SVL of 59 cm (right, white arrow: LAG). EB: Endosteal Bone; MC: Marrow Cavity.

**FIGURE 3:**
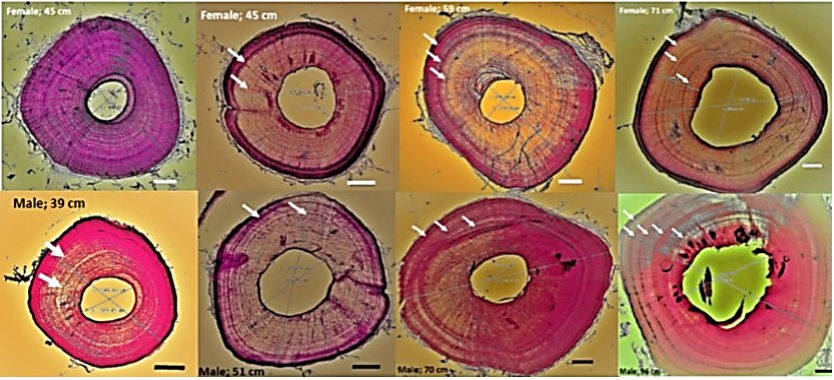
The top row shows cross sections of the female fibula, from left to right there are individuals with unclear LAG to individuals with clearly visible LAG as the SVL increases. The lower row shows cross sections of the male fibula, from left to right are young individuals with 2 LAGs to old individuals with 5 LAGs. The bar = 100 μm.

The cross-section results of the mid-diaphysis fibular bone for all individuals showed that the LAG was not always clearly visible. Individuals with an SVL of 39 cm to 70 cm showed many LAGs (Fig. 2); only a few individuals had apparent LAG. As the SVL increased (> 70 cm), the LAG became clearly visible (Fig. 3). Endosteal bones (EB) appear to be present in only a few individuals. In contrast, most of the EB fell off from the main bone, perhaps because of the long decalcification process, or the EB had been eroded due to the resorption process in the marrow cavity. The destruction of the EB leaves a smooth round shape of the marrow cavity wall; however, an undulating shape of the marrow cavity wall occurs when the EB is eroded due to the resorption process (Fig. 3).

LAGs are visible and easy to analyse in individuals with an SVL longer than 70 cm (Fig. 3). Using a wild varanid indicator as described by Andrews (1995) (Fig. 3), the estimated ages, by examining the number of LAG, of present *V. s. macromaculatus* for females with an SVL of 40.1 cm to 71.0 cm are 2–3 years old, and for males with an SVL range of 39.0 cm to 96.0 cm are 2–5 years old (Fig. 3).

**FIGURE 4:**
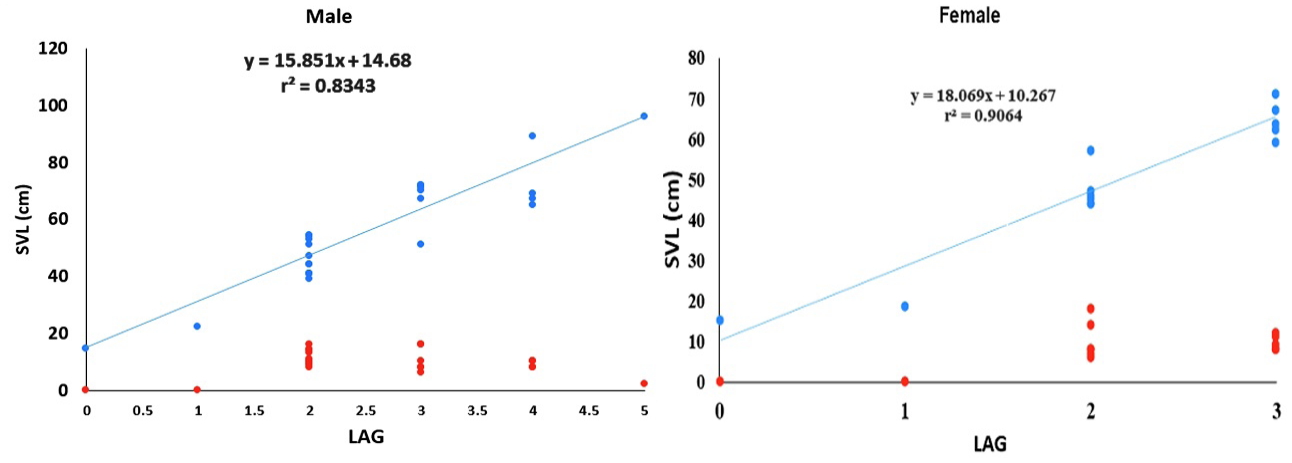
The number of LAGs (blue dots) and the number of lines (red dots) versus SVL in females and males of *V.s. macromaculatus*. SVL data for 0 LAG (female, SVL 15.1 cm; male, SVL 14.4 cm) and 1 LAG (female, SVL 18.8 cm; male, SVL 22.2 cm) (Kurniati *et al*. 2023).

In the female group, 11 (26%) individuals had visible LAGs, while in the male group, there are 22 individuals (56%). One-way ANOVA for the number of double or multiple LAGs in females and males showed no significant difference (f = 0.2976; df = 79.03; *p* = 0.5869), indicating that sexual dimorphism is not affecting the LAG appearance. The increase in SVL, which in line with the increase of LAGs formation in the SVL of 40 cm–71 cm (the female group) and 39 cm–96 cm (the male group), shows that in this phase, growth is still linear in both sexes, with a strong correlation (*r*^2^ > 0.80; Fig. 4).

**FIGURE 5:**
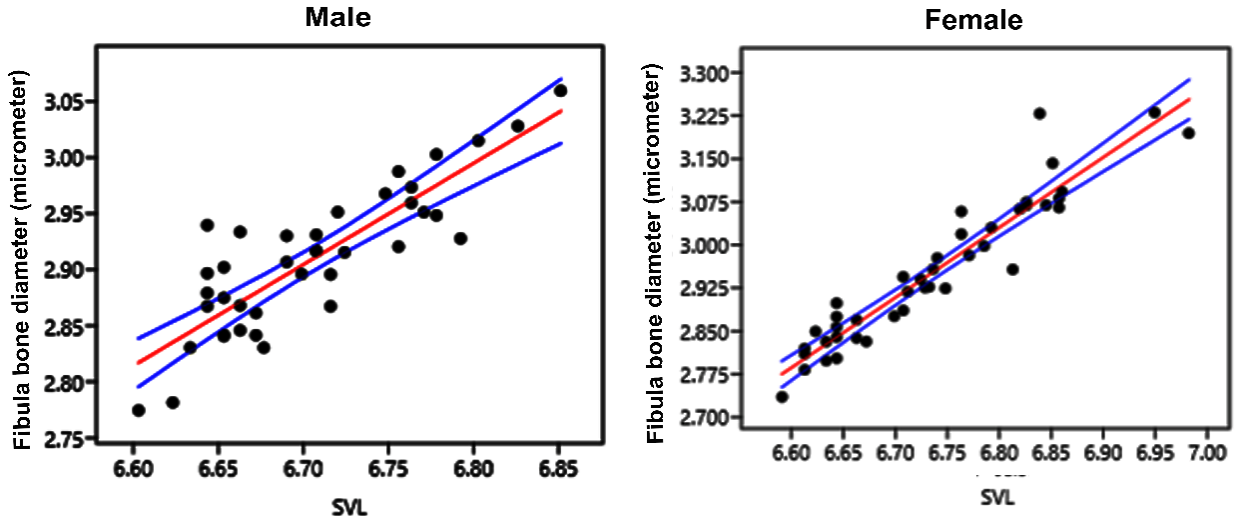
The number of LAGs (blue dots) and the number of lines (red dots) versus SVL in females and males of *V.s. macromaculatus*. SVL data for 0 LAG (female, SVL 15.1 cm; male, SVL 14.4 cm) and 1 LAG (female, SVL 18.8 cm; male, SVL 22.2 cm) (Kurniati *et al*. 2023).

Linear regression analysis showed a strong correlation between the SVL and fibular mid-diaphysis diameter in both females (*r*^2^ = 0.75; *p* = 0.0001) and males (*r*^2^ = 0.86; *p* = 0.0001) (Fig. 5, Table 1). LAGs form steadily yearly, although they are not always clearly visible in every individual, especially young individuals. The phenomenon of double and multiple LAGs visibility is that it occurs in young individuals, but only visible as single LAG in older individuals.

To test for body growth differences in the diameter of the fibula between sexes, the SVL was divided into two groups based on the SVL size, the 40 cm–50 cm and 51 cm–60 cm groups (Table 1). The result showed no significant difference between sexes for both groups (Welch F test for unequal variances: f = 4.959; df = 32.77; *p* = 0.0532 for 40 cm–50 cm SVL for female; and f = 1.16; df = 18.77; *p* = 0.3777 for 51 cm–60 cm SVL for male).

## DISCUSSION

This study indicates a shift in the minimum size of *V. s. macromaculatus* being captured in the past 30 years. Shine *et al*. (1996; 1998) found that collectors still accepting individuals with an average SVL of 32.5 cm, whilst in the current study has increased its minimum size almost double to an SVL of 51 cm–60 cm. A long SVL reaching 100 cm could still be found, as observed by Shine *et al*. (1996; 1998), but it is only in a low number and is considered priceless owing to massive skin scratch marks. The current study supports this condition.

The highest number of *V. s. macromaculatus* collected two decades ago was at SVL of 40 cm–60 cm (Shine *et al*. 1998). According to Kurniati and Riyanto (2008), the skin of *V. salvator* with a belly width between 27 cm to 40 cm is considered as grade one with a good commercial price. At this size, it coincides with the SVL of 45.59 cm–70.69 cm. Based on Shine *et al*. (1996; 1998) study, males *V. s. macromaculatus* enter early maturation at the SVL of 42.5 cm, while females at 52.5 cm. Thus, most individuals of both sexes majority have not reached the reproductive capacity to breed and produce offspring. The current minimum SVL accepted by collectors in Sumatra is at a range of 51 cm–60 cm, It seems has allowed more individuals to produce eggs and conceived in the wild. However, the success rate of these hatchlings remains unclear.

Castanet *et al*. (1993) observed that all vertebrates have annual growth marks unless they reach complete growth in less than a year. Yet, not all growth marks are yearly; they might occur haphazardly or in dense stacks inside the cortex. Although additional research is required to determine the cause of such noncyclical growth marks, they could represent a reaction to certain physiological stressors (Woodward *et al*. 2013). The primary problem observed throughout this investigation was the significant prevalence of double or multiple LAGs in both males and females.

The double LAGs represent growth interruption twice a year. It is frequently found in organisms that live in environments with many resting periods (Guarino & Erismis 2008). In this study, multiple LAGs occurred since *V. s. macromaculatus* was active over the years and the temperature was not significantly different across the year. In varanids, double or multiple LAGs also noticed in *V. griseus* (Figs. 2 and 3; Smirina & Tsellarius 1996). The LAG formed in *V. s. macromaculatus* while the growth rate remained linear and had a strong correlation (Figs. 4 and 5), indicating that *V. s. macromaculatus* is still in a fast-growing condition. Bin Abdul and Bin Abdullah (1988) also mentioned the rapid growth of *V. salvator* in Malaysia from neonates to one years old, can reach an average of SVL growth of 22 cm per year.Therefore, the zone formed in the fibula of *V. s. macromaculatus* is a LAG indicator that can be used to estimate age. The current study also shows that the number of two LAGs is not significantly different from the increase in SVL. Captive *V. salvator* in India reaches their sexual maturity in both sexes at 50 cm SVL, at the end of their second year (Andrews 1995).

Sexual dimorphism in *V. s. macromaculatus* was not visible in the body growth profile. Shine *et al*. (1998) found only a slight difference in body size between adult males and females in *V. s. macromaculatus*, in which the male head size was larger than that of the females. Du *et al*. (2014) also reported the differences in the head size of *V. salvator* in China. According to Frynta *et al*. (2010), V. indicus will show sexual dimorphism in body size (SVL) after the females have reached asymptotic growth, whereas in males, it takes longer than in females. Bennett (1998) mentioned that *V. salvator* is a Varanidae with a large body size, where individuals enter asymptotic growth after achieving optimum growth when it reaches an SVL of 160 cm (Bin Abdul & Bin Abdullah 1988).

## CONCLUSION

The visible growth mark formed on the fibula of *V. s. macromaculatus* as Line of Arrested Growth (LAG) can be used for age estimation. The estimated ages of *V. s. macromaculatus* harvested for the leather industry in South Sumatra were 2–3 years for female and 2–5 years for male. As there was no sexual dimorphism in *V. s. macromaculatus*, it shows that some of the harvested individuals have entered asymptotic growth at current SVL sizes. This shows that the harvested individuals were still in a fast-growing body stage and some might have the opportunity to lay eggs in female or conceive in the male.

## Figures and Tables

**TABLE 1:** Descriptive statistics of mid-diaphysis fibular bone diameter and linear regression equations of snout-to-vent length (SVL) group (in μm) versus the diameter of the fibula for the females and males SVL groups.

Group of individuals	Number of individuals	Mid-diaphysis Fibula bone diameter Average ± SD (range) – μm	Linear regression	*r*	*r* ^2^	*p*
Female (total sample, SVL 40–71 cm)	39	821.08 ± 122.62 (595.0 – 1147.29)	y = 0.0001x + 84.59	0.86911	0.75536	0.0001
Female, SVL 40–50 cm	20	740.34 ± 76.68 (595.0 – 870.5)	y = 0.0002x + 8.0965	0.50934	0.25943	0.0226
Female, SVL 51–60 cm	15	869.11 ± 73.03 (736.74 – 1006.44)	y = 0.0001x + 61.839	0.70929	0.50309	0.0042
Male (total sample, SVL 39–96 cm)	42	938.48 ± 289.42 (544.01 – 1701.36)	y = 0.0002x – 200.02	0.92998	0.86485	0.0001
Male, SVL 39–50 cm	16	691.73 ± 52.31 (544.01 – 792.14)	y = 0.0001x + 28.952	0.64556	0.41675	0.0065
Male, SVL 51–60 cm	12	906.93 ± 103.89 (769.3 – 1143.57)	y = 0.0003x - 661.94	0.76618	0.58704	0.0033
Male, SVL 61–70 cm	8	1169.32 ± 233.96 (906.42 – 1692.75)	y = 0.0005x – 1818.9	0.60818	0.36989	0.1005
